# Functional network alterations and their structural substrate in drug-resistant epilepsy

**DOI:** 10.3389/fnins.2014.00411

**Published:** 2014-12-11

**Authors:** Lorenzo Caciagli, Boris C. Bernhardt, Seok-Jun Hong, Andrea Bernasconi, Neda Bernasconi

**Affiliations:** Neuroimaging of Epilepsy Laboratory, McConnell Brain Imaging Center, Montreal Neurological Institute and Hospital, McGill UniversityMontreal, QC, Canada

**Keywords:** epilepsy, connectivity, resting-state, graph-theory

## Abstract

The advent of MRI has revolutionized the evaluation and management of drug-resistant epilepsy by allowing the detection of the lesion associated with the region that gives rise to seizures. Recent evidence indicates marked chronic alterations in the functional organization of lesional tissue and large-scale cortico-subcortical networks. In this review, we focus on recent methodological developments in functional MRI (fMRI) analysis techniques and their application to the two most common drug-resistant focal epilepsies, i.e., temporal lobe epilepsy related to mesial temporal sclerosis and extra-temporal lobe epilepsy related to focal cortical dysplasia. We put particular emphasis on methodological developments in the analysis of task-free or “resting-state” fMRI to probe the integrity of intrinsic networks on a regional, inter-regional, and connectome-wide level. In temporal lobe epilepsy, these techniques have revealed disrupted connectivity of the ipsilateral mesiotemporal lobe, together with contralateral compensatory reorganization and striking reconfigurations of large-scale networks. In cortical dysplasia, initial observations indicate functional alterations in lesional, peri-lesional, and remote neocortical regions. While future research is needed to critically evaluate the reliability, sensitivity, and specificity, fMRI mapping promises to lend distinct biomarkers for diagnosis, presurgical planning, and outcome prediction.

## Introduction

About 50 million people worldwide suffer from epilepsy (Kwan and Brodie, 2000). This condition is one of the most prevalent chronic neurological disorders, affecting about 1% of the general population (Leonardi and Ustun, 2002). Epilepsy is broadly characterized by recurrent spontaneous seizures resulting from an altered balance between excitation and inhibition in brain networks (Scharfman, 2007). Approximately one third of epileptic patients suffer from intractable seizures despite adequate medical treatment (Kwan et al., 2010). Patients with drug-resistant epilepsy should be promptly identified and successfully managed, as refractory seizures are associated with progressive brain damage (Cascino, 2009), devastating cognitive and socio-economic consequences (Pugliatti et al., 2007), as well as an increased risk of mortality (Mohanraj et al., 2006). The most frequent drug-resistant epilepsy syndromes are temporal lobe epilepsy (TLE) related to hippocampal sclerosis, and extra-temporal lobe epilepsy related to focal cortical dysplasia (FCD). Epilepsy surgery is recognized as the most effective treatment strategy to ensure seizure freedom (Engel et al., 2012).

The advent of structural Magnetic Resonance Imaging (MRI) has revolutionized the preoperative workup in intractable epilepsy (Koepp and Woermann, 2005; Duncan, 2010; Bernasconi et al., 2011). Furthermore, by allowing a reliable identification of the lesion giving rise to the seizures, MRI quantitative analysis lends non-invasive markers that have substantially increased the success rate of epilepsy surgery (Duncan, 2010; Bernasconi et al., 2011; Engel et al., 2013). Nevertheless, despite constant improvements in MRI acquisition and analysis technology, up to 50% of operated patients continue having seizures (McIntosh et al., 2004; Tellez-Zenteno et al., 2005; De Tisi et al., 2011). Although reasons for unfavorable results are not fully understood, emerging imaging data suggest that anomalies extending beyond the lesion may negatively impact outcome (Keller et al., 2007; Bernhardt et al., 2010, 2011; Voets et al., 2011; Bonilha et al., 2013). These observations challenge the conventional model of “focal epilepsy” and revive the concept of distributed neural networks (Spencer, 2002; Richardson, 2012).

**Figure 1:**
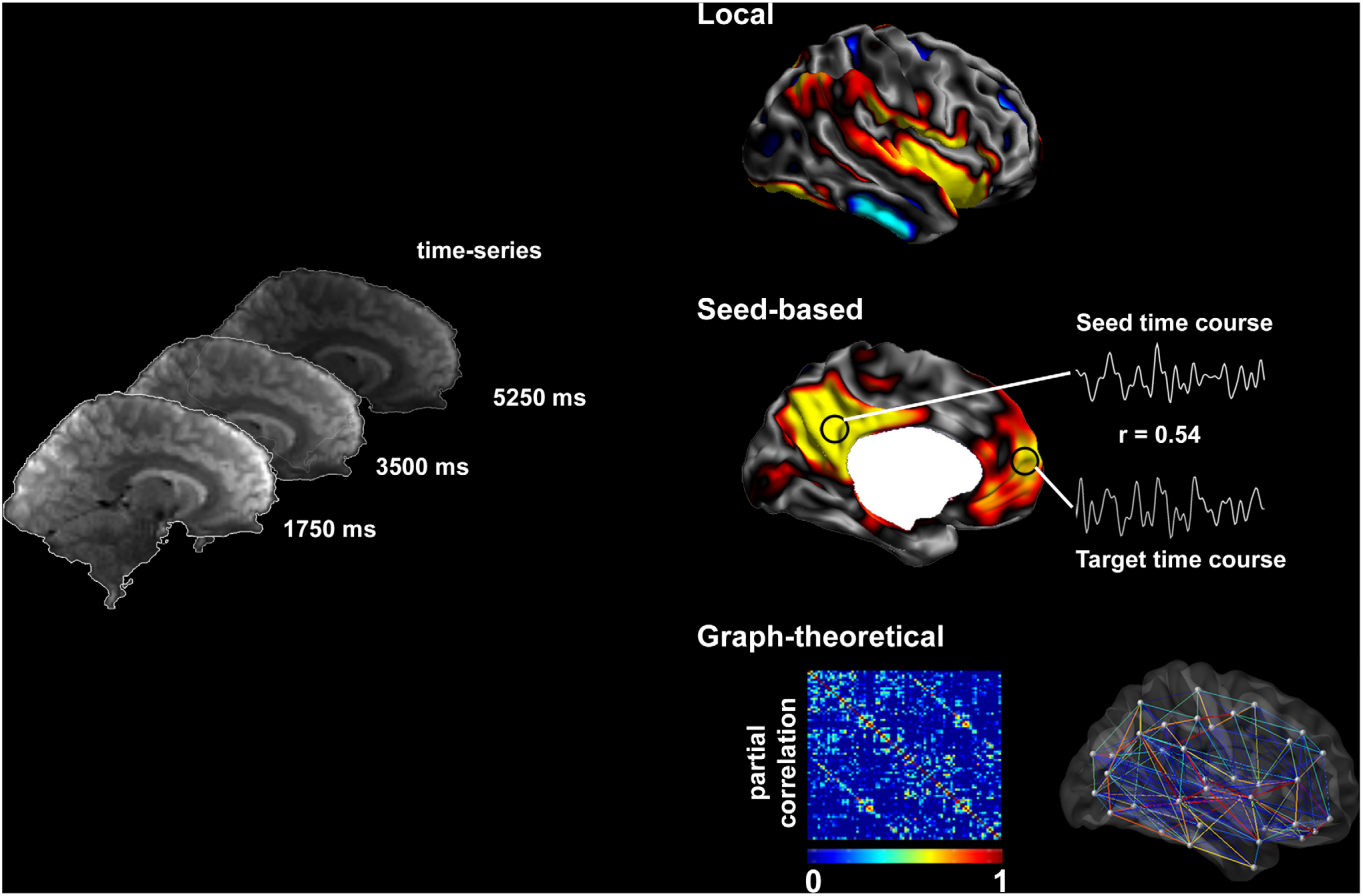
**Methods to assess resting-state brain function**. Resting-state fMRI time series allow the description of functional networks at multiple levels. Local markers of functional integrity can be derived from the amplitude of low-frequency fluctuations. Seed-based analysis of connectivity relies on cross-correlations between time-series of seed and target regions. Systematic seeding across multiple regions allows for the generation of connectivity matrices and equivalent connectivity graphs; these can, in turn, be analyzed using graph-theory to address large-scale network topology.

Advances in non-invasive neuroimaging techniques allow probing connectivity *in vivo*. While physical properties of structural brain networks can be derived from diffusion MRI, functional techniques (such as functional MRI and magnetoencephalography) model connectivity as statistical dependencies of neurophysiological time series (Biswal et al., 1995; Srinivasan et al., 2007; Friston, 2011). Functional MRI (fMRI) utilizes changes in blood oxygen level-dependent (BOLD) signal to infer neuronal activity (Logothetis et al., 2001). The link is understood under a neurovascular coupling model: neuronal activity in a region leads to increased blood flow to supply oxygen and nutrients. The vascular response leads to a biomagnetic perturbation of susceptibility, which is detected by T2^*^ sequence used for BOLD fMRI. Conventionally, fMRI has a relatively coarse time-resolution (order of seconds; but see Feinberg et al., 2010), but good spatial-resolution and whole-brain coverage. In drug-resistant epilepsy, most earlier studies have used task-related fMRI to map brain activation of eloquent areas, mainly those subserving language, memory (Berl et al., 2005; Janszky et al., 2005; Voets et al., 2009), and sensory-motor function (Janszky et al., 2003; Jirsch et al., 2006; Dumoulin et al., 2007; Sommer et al., 2013). Recent advances focus on spontaneous modulations in BOLD signal that occur during “resting” (i.e., task-free) conditions (Figure 1) (Fox and Raichle, 2007; Van Essen et al., 2012; Cabral et al., 2014). Advantages over task-related paradigms include the possibility to examine multiple cortical areas in one session, minimal demands on patients with reduced ability to perform tasks, and the possibility to aggregate data across sites. Resting-state networks are highly reproducible across subjects (Damoiseaux et al., 2006; Biswal et al., 2010; Cabral et al., 2014) and have been shown to correspond closely to brain systems engaging in specific tasks (Greicius et al., 2003; Fox et al., 2006; Smith et al., 2009).

**Figure 2:**
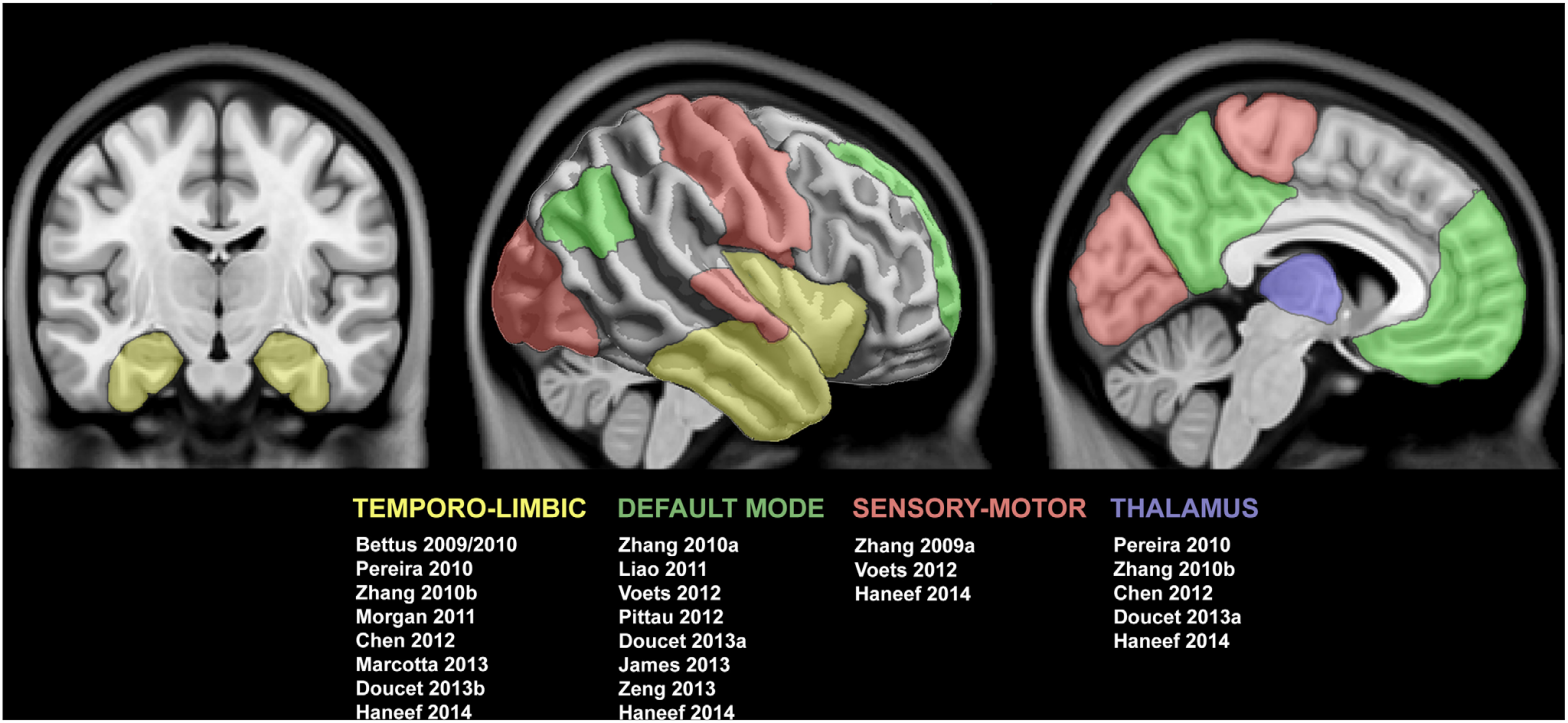
**Summary of studies reporting functional connectivity anomalies in temporal lobe epilepsy**. Cortical and subcortical regions displaying functional alterations are color-coded with respect to the associated network. See text for details.

In this review, we will principally focus on TLE, and outline the available evidence of functional anomalies spanning from limbic circuits to whole-brain networks. We will also detail preliminary findings on functional disruptions in FCD, although studies on patients with this condition are relatively sparse and cohorts often inhomogeneous. We will discuss how functional alterations could be related to those observed in structural MRI. Finally, we will critically evaluate whether and how fMRI measures could serve as effective biomarkers for the pre-surgical workup in drug-resistant epilepsy. Figure 1 provides a schematic overview of the methods to assess resting-state brain function and Figure 2 summarizes findings in TLE.

## The study of functional networks using resting-state fMRI

Recent years have witnessed a dramatic increase in resting-state fMRI analyses to probe intrinsic functional networks in healthy and diseased populations (Biswal et al., 1995, 2010; Greicius et al., 2003; Smith et al., 2009). In most studies, the participant lies still in the scanner for 5–7 min, a scan-time that was previously shown to deliver stable connectivity measures (Van Dijk et al., 2010). More recent work suggests that reliability can be further improved when with longer duration scans of 9–12 min (Birn et al., 2013), and work focusing on individual subject classification suggests even longer acquisitions (Anderson et al., 2011). There is some evidence for variable effects of eye opening (with or without fixation) or closure on connectivity measures, possibly hampering replication of findings (Yan et al., 2009; Patriat et al., 2013).

Reflecting the growing importance of resting-state acquisitions, numerous software packages have been developed for automatic data processing (e.g., Yan and Zang, 2010). Conventional processing includes the discarding of a few time points at the onset of the recording to ensure steady-state magnetization, followed by correction for subject motion through linear registration of individual time points to a reference time point, as well as co-registration between structural and functional images. These basic operations are often followed by statistical correction for subject-motion and average signal from the white matter and cerebro-spinal fluid as a proxy for physiological activity. While most early resting-state studies corrected for global signal, the validity of this preprocessing step is currently controversially discussed (Fox et al., 2009; Murphy et al., 2009). More recent work invokes *scrubbing*, an approach that models time points affected by heavy motion as additional nuisance regressors (Power et al., 2012). Corrected time-series are filtered, mostly to a pass-band close to 0.1–0.01 Hz, and often subsequently mapped to individual cortical surface models and/or a standard stereotaxic space for group-level inference. Analytical approaches include seed-based functional connectivity assessments, data-driven approaches that cluster the brain into regional assemblies showing similar functional activations such as independent component analysis (ICA), the extraction of voxel-based regional markers, and graph theoretical assessments to study topology at large scale.

Previous work in healthy individuals has shown that resting-state fMRI networks are generally reproducible across subjects (Damoiseaux et al., 2006), show appropriate test-retest reliability (Shehzad et al., 2009), and may closely correspond to brain systems engaging in specific tasks (Biswal et al., 1995; Smith et al., 2009; Laird et al., 2011; Tusche et al., 2014). Studies in primates have suggested a close correspondence between intrinsic functional connections and anatomical pathways derived from tract tracing (Margulies et al., 2009; Mantini et al., 2011; Shen et al., 2012). Analysis of resting-state patterns may furthermore help to subdivide specific anatomical regions (Margulies et al., 2007; Mars et al., 2011; Steinbeis et al., 2014). Finally, resting-state connectivity may be altered in disease conditions (Greicius, 2008; Fox and Greicius, 2010; Kelly et al., 2012).

## Functional network disruptions in TLE: limbic and peri-limbic connectivity

The majority of resting-state fMRI work in TLE addressed the functional connectivity of limbic structures through seed-based analysis. Impaired connectivity has been consistently detected within mesiotemporal structures ipsilateral to the seizure focus; the most prominent local alterations involve the links between rostral and caudal hippocampus, and between the rostral hippocampus and the enthorinal cortex (Bettus et al., 2009, 2010). Reduced functional connectivity is observed between ipsilateral and contralateral hippocampi (Pereira et al., 2010; Morgan et al., 2011; Maccotta et al., 2013), as well as between the epileptogenic mesial temporal structures and bilateral lateral temporal neocortices (Pereira et al., 2010; Maccotta et al., 2013; Doucet et al., 2013a). Left TLE patients seem to display more marked connectivity alterations than those with right TLE, both in the epileptogenic hemisphere and in contralateral limbic areas (Pereira et al., 2010). Breakdowns in ipsilateral functional connectivity may co-occur with connectivity increases in contralateral mesiotemporal networks, which have been shown to positively correlate with working memory performance (Bettus et al., 2009, 2010). Such increases may reflect reorganization of limbic networks. Supporting evidence for abnormal local functional connectivity within peri-limbic regions comes from a preliminary observation of enhanced amplitude of the low-frequency fluctuations of BOLD signal, a local functional marker possibly reflective of long-range neuronal synchronization (Balduzzi et al., 2008), in the ipsilateral mesiotemporal structures and lateral temporal neocortex (Zhang et al., 2010b).

The spatial patterns of resting-state functional alterations seem to parallel the structural damage identified by quantitative MRI analysis. In agreement with histopathological studies, atrophy has been confirmed beyond the hippocampus to include the enthorinal cortex and the amygdala complex (Bernasconi et al., 1999, 2001b, 2003; Salmenperä et al., 2000; Bartolomei et al., 2005), with evidence for subregional disease progression (Briellmann et al., 2002; Fuerst et al., 2003; Bernasconi et al., 2005; Bernhardt et al., 2013b). Besides, morphological disruptions have been identified in the perirhinal, temporo-polar, and lateral temporal neocortices ipsilateral to the focus (Jutila et al., 2001; Moran et al., 2001; Coste et al., 2002; Bernasconi et al., 2004; Sankar et al., 2008; Voets et al., 2011). Interestingly, we detected increased cortical folding complexity, which involved the contralateral temporo-polar region in right TLE (Voets et al., 2011). Differently from the cognitively adaptive functional reorganization (Bettus et al., 2009, 2010), the contralateral increase in cortical folding predicted unfavorable post-surgical outcome (Voets et al., 2011).

Further evidence for limbic and peri-limbic disruptions comes from structural connectivity studies, which have employed either diffusion imaging or structural MRI covariance (Bernhardt et al., 2013a). Diffusion imaging constitutes a versatile tool to assess the microstructural integrity of the white matter, and to investigate its architecture through the reconstruction of fiber pathways. Fractional anisotropy, indicating the extent to which water diffusion deviates from a random spherical model, is consistently decreased in temporo-limbic tracts, such as the fornix, the cingulum (Ahmadi et al., 2009; Concha et al., 2009), and the uncinate fasciculus (Rodrigo et al., 2007; Diehl et al., 2008; McDonald et al., 2008). Mean diffusivity, a scalar measure of overall diffusivity, appears markedly altered in the proximity of the epileptogenic zone (Focke et al., 2008; Concha et al., 2009, 2012). Structural MRI covariance analysis relies instead on inter-regional correlations of structural markers, such as cortical thickness or gray matter volume, to infer network properties (Alexander-Bloch et al., 2013). We and others (Bonilha et al., 2007; Bernhardt et al., 2008) have shown decreased structural coordination between mesiotemporal regions and lateral temporal neocortices. These results parallel functional connectivity derangement within the temporo-limbic circuits (Maccotta et al., 2013).

## Functional disruptions in TLE: involvement of widespread brain networks

Several studies have identified abnormal connectivity patterns between seeds placed within the epileptogenic mesiotemporal region and bilateral clusters in the posterior cingulate cortices, precuneus, inferior parietal lobules and mesial prefrontal cortices (Pittau et al., 2012; James et al., 2013; Doucet et al., 2013b; Haneef et al., 2014). Along with the hippocampi and the parahippocampal gyri, this set of regions constitutes the default mode network (DMN), a system putatively involved in internally-focused activities including memory retrieval, mind wandering and envisioning the future (Buckner et al., 2008; Christoff et al., 2009). Functional connectivity disruptions in the DMN have been elucidated in TLE patients also by studies extracting resting-state networks via ICA (Zhang et al., 2010a; Liao et al., 2011; Voets et al., 2012), placing seeds in non-temporal DMN areas (Haneef et al., 2012), or assessing regional homogeneity of resting fMRI time-courses (Zeng et al., 2013). Prominent DMN alterations in TLE could be explained in view of the relevance of the hippocampi in this resting-state network (Buckner et al., 2008). As a complementary finding, EEG-fMRI analyses have also shown dysfunction in relation to epileptic spikes in areas pertaining to the DMN (Kobayashi et al., 2006; Laufs et al., 2007). The extent to which left and right TLE patients differ with respect to DMN connectivity is not clear: some studies did not identify substantial differences (Pittau et al., 2012), while others reported greater functional disconnectivity in left (James et al., 2013; Doucet et al., 2013b; Haneef et al., 2014) or right TLE (Zhang et al., 2010a; Haneef et al., 2012; Voets et al., 2012). Inconsistencies regarding the extent of DMN abnormalities and divergences with regard to seizure focus could be ascribed to methodological discrepancies among studies, such as procedures involved in network extraction (e.g., seed-based vs. ICA-based approaches), statistical thresholding and differences across patient cohorts, particularly in relation to pharmaco-response. Resting-state fMRI analyses, both ICA and seed-based, have also detected connectional disruptions in areas involved in primary sensory processing (Zhang et al., 2009a; Voets et al., 2012; Haneef et al., 2014) and attention (Zhang et al., 2009b). Furthermore, impaired functional interactions are reported between the epileptogenic mesiotemporal lobes and subcortical areas, including the thalamus and the brainstem (Pereira et al., 2010; Pittau et al., 2012; Doucet et al., 2013b; Haneef et al., 2014).

Addressing the relationship between connectivity and cognitive performance, Waites et al. (2006) described altered functional signaling at rest in middle and inferior frontal as well as cingulate regions in patients with left TLE (Waites et al., 2006). Their findings might represent an intrinsic functional correlate of the subtle language disturbances often identified in this group (Hermann et al., 1997). In left TLE, increased functional interactions between epileptogenic mesial temporal structures and the ipsilateral posterior DMN appear to be associated with poorer verbal memory abilities (Doucet et al., 2013a; Holmes et al., 2014), while increased coupling between the ipsilateral hippocampus and contralateral posterior DMN shows a positive relation with improved verbal memory (Holmes et al., 2014). In right TLE, strengthening of connections between the left mesial temporal lobe and ROIs in the ipsilateral mesial prefrontal cortex predicted better non-verbal memory (Doucet et al., 2013a). This suggests that functional reorganization involving the recruitment of contralateral areas might represent a compensatory phenomenon favoring cognitive performance.

A number of seed-based studies have sought for possible functional correlates of psychiatric comorbidities in relation to depression. Derangements in functional connectivity between mesiotemporal lobes and prefrontal cortices might occur in depressed TLE patients (Chen et al., 2012; Kemmotsu et al., 2013). Compared to non-depressed subjects, depressed patients seem also to exhibit increased functional coupling between the limbic system and the angular gyrus, possibly suggestive of an intensified susceptibility to environmental cues (Chen et al., 2012). Furthermore, there is preliminary evidence that maladaptive reorganizations of functional connections between bilateral amygdalae and prefrontal (Kemmotsu et al., 2013), lateral temporal cortex and the cuneus (Doucet et al., 2013b) might relate to depressive and anxiety symptoms.

Evidence for functional disruptions in multiple brain networks in TLE confirms and expands the literature on extra-temporal structural abnormalities. Extensive neocortical anomalies have been pinpointed in several volumetric and cortical thickness analyses (Bernasconi et al., 2004; Lin et al., 2007; Bernhardt et al., 2008, 2009b; Keller and Roberts, 2008; Mueller et al., 2009), and gray matter loss has been observed in subcortical structures, including the thalamus and basal ganglia (Dreifuss et al., 2001; Natsume et al., 2003; Bernhardt et al., 2012). Structural connectivity studies employing diffusion imaging have demonstrated decreased fractional anisotropy in a consistent set of white matter tracts, including the inferior and superior longitudinal fascicles (Focke et al., 2008; Lin et al., 2008; Ahmadi et al., 2009), the internal and external capsule, and the corpus callosum (Arfanakis et al., 2002; Gross et al., 2006; Concha et al., 2009). On the other hand, disruptions in mean diffusivity seem to be relatively less extended (Concha et al., 2005; Focke et al., 2008). Our group has recently shown that diffusivity values normalize as a function of the anatomical distance from the seizure focus (Concha et al., 2012). Structural covariance analyses have described abnormal correlations between mesiotemporal regions and a variety of areas, including pre-frontal, fronto-central, cingulate and occipito-temporal neocortices (Bonilha et al., 2007; Bernhardt et al., 2008; Mueller et al., 2009). We demonstrated that thalamic atrophy co-varies with cortical thickness of mesiotemporal, fronto-central and lateral temporal cortices (Bernhardt et al., 2012).

Although there are similarities in the location of functional and structural abnormalities, systematic assessments of the relationship between changes in both domains are scarce. On the one hand, it is not very well understood whether more gray matter in a certain region relates to stronger functional activation, or changes in functional connectivity. On the other hand, patients may express significantly higher structural variability of certain brain regions than controls. This may possibly impact the quality of across-subjects alignment during preprocessing differentially in both groups, particularly when conventional group-level analyses are carried out in a standard voxel space. Functional analysis in subject-specific space, ideally on anatomy-informed models of the cortex, may control for some of these confounds. While multi-modal imaging could shed light on structure-function relationships in epilepsy, few studies have directly addressed this question. For instance, a study showed that impaired functional connectivity between mesial temporal lobes and posterior cingulate cortex correlated with reduced white matter density of bundles connecting the two regions (Liao et al., 2011). Using whole-brain analysis, our group found disruptions in functional connectivity between mesiotemporal regions and neocortical areas, including regions in the DMN and sensory-motor networks. Importantly, functional connectivity changes of the hippocampus were partially explained by gray matter density estimates of this region, suggesting that altered signal coupling may reflect hippocampal damage. Moreover, functional connectivity changes outside of mesiotemporal region correlated with diffusion parameters inter-connecting fiber tracts (Voets et al., 2012). This lead us postulate that morphological and architectural derangements account for alterations in intrinsic functional connectivity in TLE.

## Evidence of disrupted network topology in TLE: insights from graph theory

Although there is significant support for local and inter-regional connectivity disruptions in TLE, the above-discussed analyses have not characterized organizational properties of brain networks. In this context, graph theoretical analysis provides a unique framework to quantify whole-brain network topology (Bullmore and Sporns, 2009; Bassett and Gazzaniga, 2011). Networks can be modeled as collections of nodes, corresponding to brain regions, which are interconnected via links (or edges). Nodal selection exerts a crucial influence on graph-theoretical parameters (Zalesky et al., 2010), and several investigations have aimed at improving the reliability of parcellation techniques (Geyer et al., 2011; Glasser and Van Essen, 2011). Network edges can be derived from both structural and functional connectivity datasets, as shown by the variety of graph-theoretical analyses relying on electrophysiology (Ponten et al., 2007), fMRI (Salvador et al., 2005), diffusion MRI (Gong et al., 2009), and structural MRI covariance (He et al., 2007). While segregation measures refer to the existence of tightly interconnected nodes within the network, known as *clusters* or *modules*, their integration is mediated via interconnecting *paths* (Bullmore and Sporns, 2009). Centrality measures are employed to identify *hubs*, i.e., nodes with a high degree of connections (Van Den Heuvel and Sporns, 2011). The global topology of brain networks in healthy individuals exhibits a *small-world* organization (Bullmore and Sporns, 2009). This architecture, which has been consistently shown across various imaging modalities, enables both segregation and integration of information processing while being maximally efficient in terms of wiring costs.

In TLE, only a few studies performed graph-theoretical analyses on functional (Liao et al., 2010; Wang et al., 2014) or structural (Bernhardt et al., 2011; Bonilha et al., 2012; Liu et al., 2014) MRI datasets. Deriving brain networks from resting-state fMRI measures, a study reported decreased clustering and path length, and disruptions in the distribution of network hubs, in favor of a random network topology (Liao et al., 2010). Conversely, a more recent study showed increased clustering and path length, a finding rather typical of a regularized topology (Wang et al., 2014). Interestingly, the latter findings are in line with our graph-theory analysis of structural networks constructed from cortical thickness correlations (Bernhardt et al., 2011), with graph-theoretical studies on diffusion MRI data (Bonilha et al., 2012; Liu et al., 2014) and with electrophysiology-derived network analyses (Bartolomei et al., 2013). Preliminary evidence suggests that alterations in brain structural (Bernhardt et al., 2011) and functional (Wang et al., 2014) networks intensify over time. We have shown that patients with a poor outcome after surgery exhibit more pronounced network disruptions compared to those who achieved seizure freedom. These findings suggest that whole-brain network analysis might be a valuable asset for clinical decision-making (Bernhardt et al., 2011).

## Focal cortical dysplasia: evidence for widespread extra-lesional abnormalities

Focal cortical dysplasia (FCD) is an epileptogenic malformation of cortical development resulting from localized abnormalities in neuronal migration and organization (Barkovich et al., 2012). Neocortical epilepsy secondary to FCD accounts for approximately half of pediatric patients and a quarter of adult subjects (Lerner et al., 2009; Bernasconi et al., 2011). Cortical dysplasias encompass a wide spectrum of histopathological changes related to cortical disorganization, including isolated dyslamination typical of FCD type I, and more severe lesions characterized by dyslamination and cytological abnormalities such as dysmorphic cells or balloon cells in FCD type II (Blumcke et al., 2011). Associated alterations in the subcortical white matter adjacent to the lesion are also frequently observed in pathological specimens (Andres et al., 2005; Sisodiya et al., 2009). The degree of histopathological disruptions influences lesional visibility on structural MRI (Lerner et al., 2009; Bernasconi et al., 2011). In this regard, patients with FCD type II display a significantly wider spectrum of MRI abnormalities compared to those with FCD type I, of whom the vast majority shows unremarkable routine MRI (Tassi et al., 2002; Krsek et al., 2008).

In recent years, MRI processing has allowed for an increased detection of subtle dysplasias (Bernasconi et al., 2001a; Antel et al., 2003; Wilke et al., 2003; Huppertz et al., 2005; Srivastava et al., 2005; Colliot et al., 2006a; Besson et al., 2008; Hong et al., 2014). Morphological anomalies, including increased gray matter density and sulcal depth may be found in areas remote from the dysplastic cortex (Bonilha et al., 2006; Colliot et al., 2006b; Besson et al., 2008). We recently employed surface-based multivariate pattern recognition to automatically detect FCD type II, and showed that 50% of patients presented at least one extra-lesional cluster characterized by abnormal sulcal morphology (Hong et al., 2014). Whole-brain diffusion imaging studies have shown evidence for peri-lesional abnormalities in the subcortical white matter contiguous to the dysplastic cortex (Lee et al., 2004; Gross et al., 2005; Widjaja et al., 2007, 2009; Diehl et al., 2010) and at distance (Eriksson et al., 2001; Guye et al., 2007; Fonseca Vde et al., 2012).

To date, relatively few fMRI studies have probed the integrity of functional networks in FCD. Assessing various malformations of cortical development, a study reported impaired activation of dysplasias located in language areas (Vitali et al., 2008). Other task-related fMRI studies assessing language in a variety of cortical malformations, have shown that disruptions may not be limited to the lesional cortex, with evidence for intra- and inter-hemispheric redistribution of function (Janszky et al., 2003; Yuan et al., 2006; Gaillard et al., 2007; Mbwana et al., 2009). Location of the lesions may have a differential impact on the expression of language dominance (Duke et al., 2012). In heterogeneous populations of non-operated adults and children with focal epilepsy and presumed dysplasia, functional connectivity disruptions have been detailed in language networks (Vlooswijk et al., 2010) and in a wide set of intrinsic functional networks (Luo et al., 2011; Widjaja et al., 2013). Additional indications of widespread functional disruptions may come from EEG-fMRI studies, which showed that spike-related BOLD signal changes occur in brain areas distant from the putative seizure onset zone, suggestive of diffuse epileptogenic networks (Federico et al., 2005; Tyvaert et al., 2008; Thornton et al., 2011). A graph-theoretical study in adults with MRI-negative focal epilepsy reported decreased global network efficiency, together with reductions in network clustering, indicative of a reorganized topology relative to controls (Vlooswijk et al., 2011). In more recent work in children with non-lesional frontal lobe epilepsy, the same group has suggested that patients present with a more regular global topology than typically developing children (Vaessen et al., 2013, 2014). It is tempting to interpret these findings in light of the fine-tuning in global network properties taking place during brain maturation (Fair et al., 2009; Dosenbach et al., 2010), which could account for shifts in brain topology across lifespan.

## Conclusions

The advent of functional mapping techniques has substantially advanced our knowledge of brain connectivity in drug-resistant epilepsy. In TLE, a growing body of evidence indicates marked connectional derangements primarily in limbic circuits, but also across multiple networks, together with profound shifts in global network topology. FCD may also be associated with complex connectional reconfigurations, both locally and at a whole-brain level, although the literature is rather sparse and patient groups are frequently inhomogeneous. On the whole, current findings suggest that these focal epilepsy syndromes may be interpreted as disorders of distributed networks in both structural and functional domains.

An important avenue for future research will be to advance our understanding of how functional connectivity relates to brain structure. Studies in healthy controls have provided evidence for substantial overlap (Honey et al., 2007; Skudlarski et al., 2008; Greicius et al., 2009). In focal epilepsy, although impairments in resting-state functional coupling seem to parallel morphological disruptions unveiled by structural MRI, very few multi-modal imaging studies specifically addressed this issue so far (Liao et al., 2011; Voets et al., 2012). Importantly, causal links between changes in both domains have not been addressed. The putative polysynaptic features of functional coupling across the brain occasionally allow for the detection of functional connectivity in the absence of direct structural connections (Uddin et al., 2008; Honey et al., 2009; Lu et al., 2011), and this complicates the interpretation and the evaluation of accuracy. Another avenue for future research is the assessment of possible variations in intrinsic connectivity over time. So far, despite ample evidence for progressive structural damage (Bernasconi and Bernhardt, 2009; Cascino, 2009; Bernhardt et al., 2009a), the ability of functional markers to track disease progression is unclear. A first assessment suggested that ipsilateral and contralateral hippocampal functional connectivity alterations might undergo variable trajectories throughout the course of the disease (Morgan et al., 2011). Combined longitudinal analysis of structure and function in clinically well characterized groups of newly diagnosed patients, particularly those with acquired conditions such as post-traumatic epilepsy may shed light on seizure-related alterations vs. those related to the epileptogenic process.

Due to their relative accessibility and ability in unveiling functional disruptions, resting-state fMRI has strongly impacted the neuroimaging community. In epilepsy, preliminary results suggest a promising role for this technique to provide biomarkers for the diagnosis, pre-surgical planning, and prediction of surgical outcome (Bettus et al., 2010; Negishi et al., 2011; Castellanos et al., 2013). Despite some evidence in psychiatric conditions for medication affecting intrinsic networks, both in the context of treatment (Schmidt et al., 2013) drug abuse (Kelly et al., 2011), there are currently no reliable predictors of drug-response and monitoring of drug-related side effects in epilepsy (Koepp, 2014). A number of caveats need to be addressed. Firstly, it is paramount to evaluate and likely improve the reliability (Castellanos et al., 2013; Fiecas et al., 2013) and validity of functional markers, given potentially profound influences of artifacts and preprocessing choices on results (Birn et al., 2006; Niazy et al., 2011; Power et al., 2012; Buckner et al., 2013). A further prerequisite will be the evaluation of sensitivity and specificity; only few studies have systematically addressed this issue in patients in the context of focus (Bettus et al., 2010; Zhang et al., 2010b; Chiang et al., 2014) and language lateralization based on resting-state fMRI data (Doucet et al., 2014). Analyzing clinically well-defined patient cohorts and cross-site assessment of reproducibility will be important to determine the clinical applicability of resting fMRI. To address the complex pathophysiology and individual susceptibilities future approaches will likely require a combination of quantitative functional and structural imaging modalities to generate biomarkers that operate at various stages of the epileptogenic process.

### Conflict of interest statement

The authors declare that the research was conducted in the absence of any commercial or financial relationships that could be construed as a potential conflict of interest.
